# Current Advances in CETSA

**DOI:** 10.3389/fmolb.2022.866764

**Published:** 2022-06-09

**Authors:** Tuomas Aleksi Tolvanen

**Affiliations:** ^1^ Division of Rheumatology, Department of Medicine Solna, Karolinska University Hospital and Karolinska Institute, Stockholm, Sweden; ^2^ Pelago Bioscience AB, Solna, Sweden

**Keywords:** CETSA, PISA, TPP, mass spectrometry, target deconvolution, target engagement

## Abstract

Knowing that the drug candidate binds to its intended target is a vital part of drug discovery. Thus, several labeled and label-free methods have been developed to study target engagement. In recent years, the cellular thermal shift assay (CETSA) with its variations has been widely adapted to drug discovery workflows. Western blot–based CETSA is used primarily to validate the target binding of a molecule to its target protein whereas CETSA based on bead chemistry detection methods (CETSA HT) has been used to screen molecular libraries to find novel molecules binding to a pre-determined target. Mass spectrometry–based CETSA also known as thermal proteome profiling (TPP) has emerged as a powerful tool for target deconvolution and finding novel binding partners for old and novel molecules. With this technology, it is possible to probe thermal shifts among over 7,000 proteins from one sample and to identify the wanted target binding but also binding to unwanted off-targets known to cause adverse effects. In addition, this proteome-wide method can provide information on the biological process initiated by the ligand binding. The continued development of mass spectrometry labeling reagents, such as isobaric tandem mass tag technology (TMT) continues to increase the throughput of CETSA MS, allowing its use for structure–activity relationship (SAR) studies with a limited number of molecules. In this review, we discussed the differences between different label-free methods to study target engagement, but our focus was on CETSA and recent advances in the CETSA method.

## Introduction

In recent years, many new drug leads have been found from phenotypic screenings, creating a demand for target deconvolution and confirmation of lead molecules' target engagement (Kaur et al., 2018; Friman, 2020; Mateus et al., 2021). The most used label-free techniques using intact cells or cell lysates are stability of proteins from rates of oxidation (SPROX) (West et al., 2008; Strickland et al., 2013), drug affinity responsive target stability (DARTS) (Lomenick et al., 2009), limited proteolysis (LiP) (Feng et al., 2014), and cellular thermal shift assay (CETSA^®^) (Martinez Molina et al., 2013) with all its variations. After the target engagement has been confirmed, the next step in the drug development pipeline is lead optimization, which is usually performed on medium- or high-throughput platforms utilizing purified protein domains or cell lysates. All the aforementioned tools can be utilized to some extent also in lead optimization, but the throughput of NanoBRET and CETSA are best suited for this step. Also, an advantage is that both can be performed in a biologically relevant environment, i.e., in intact cells. Only CETSA can be used in the *in vivo* test phase to ensure proper *in situ* target engagement in the tissues and to investigate possible off-targets. All the methods have their own advantages and disadvantages and should be considered to complement each other instead of competing against each other. This review introduces shortly the differences of these aforementioned methods, however, with a focus on CETSA and its applications, as well as together with other methods.

Applications of CETSA are not limited to drug discovery; even this review focused on those. CETSA can be used, for example, for studying evolutionary biology in the tree of life (Jarzab et al., 2020) or to find novel thermostable enzymes for industrial applications (Oztug et al., 2020). As CETSA detects changes in protein, unfolding it could detect novel biomarkers or drug targets for some diseases; even the disease would not alter the protein amounts or the expression pattern.

## 2 Different Label-Free Cell-Based Methods for Target Engagement

CETSA is based on the traditional thermal shift assay (TSA) which detects a change in protein thermal stability induced by a ligand binding. The most profound difference between these two methods is that TSA is performed for individual purified recombinant proteins or isolated protein domains, whereas CETSA is performed with whole cells or cell lysates. The method was developed at Karolinska Institutet, Sweden and Nanyang Technological University, Singapore, and was first published in 2013 (Martinez Molina et al., 2013). The proteins’ thermal shift was detected using Western blotting in the original publication (Martinez Molina et al., 2013), but soon thereafter, bead-based (Jarafi et al., 2014) and mass spectrometry detection (Savitski et al., 2014) methods were also published. CETSA using the bead-based chemistry detection method is primarily used in high-throughput applications. Both Western blotting and CETSA HT require high-quality antibodies which are not always available for all the interesting targets. CETSA utilizing mass spectroscopy as a detection method is referred to in the literature as CETSA MS, thermal proteome profiling (TPP), or proteome integral solubility alteration (PISA). The principles and differences between these methods will be discussed later in this review.

**FIGURE 1:**
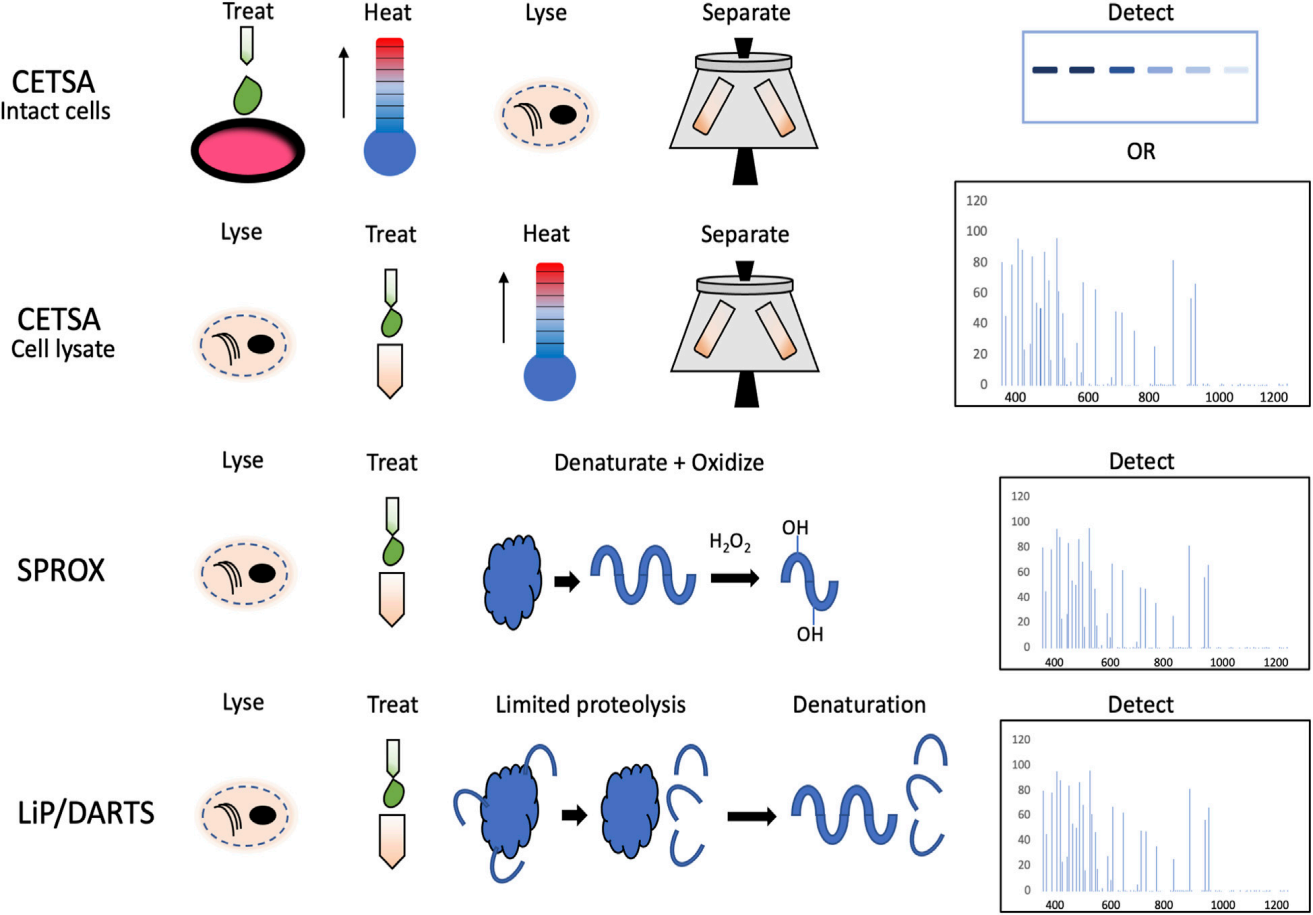
Schematic overview of the workflows in CETSA performed on intact cells and cell lysates, SPROX, and limited proteolysis (LiP).

The principles of SPROX, DARTS, and LiP with their advantages and limitations have been reviewed thoroughly by Kaur et al. (2018). In brief, all these methods are based on the principle that ligand binding causes changes to protein conformation. In DARTS and LiP, the samples are treated with protease which cannot access the same cleavage sites on the ligand-bound protein as on the protein without the ligand treatment. These differently spliced peptides from the same protein are then detected with MS. In SPROX, the samples are subjected to a chemical denaturant gradient together with an oxidizing agent. Differential protein unfolding can be detected by highly specific methionine oxidation patterns of the peptides. The advantage of DARTS/LiP and SPROX is that the data they provide are not limited to the level of affected proteins. They also provide information about the potential binding site of the ligand which CETSA cannot provide. The pitfall of the methods is that they rely heavily on the data obtained from one peptide which increases the number of false positives and requires cell lysis before the treatment with a compound (Kaur et al., 2018). The difference in the workflows between afore mentioned methods and CETSA is illustrated in Figure 1.

NanoBRET differs the most from the aforementioned methods as it requires a cell line expressing luciferase-tagged target protein and a substrate for the luciferase. The ligand binding to the target protein changes the protein conformation which decreases the luciferase activity (Dale et al., 2019). Scientists from Merck have developed a nano luciferase detection system for CETSA called HiBiT thermal shift assay (BiTSA) (Mortison et al., 2021). BiTSA combines the CETSA and parts of NanoBRET to create an antibody-free high-throughput detection system. It is based on a split luciferase system in which the 11-amino-acid peptide tag, HiBiT, is connected to the target protein using the CRISPR-CAS9 system and a complementary partner LgBiT added to the experimental system after heating. When in complex together HiBiT-LgBiT produces bioluminescence, but when the target protein is denatured, LgBiT cannot bind to HiBiT. The luciferase signal attenuates linearly together with the amount of ligand bound to the target protein (Dale et al., 2019; Mortison et al., 2021). NanoBRET and BiTSA both require an engineered cell system to produce a tagged target protein but can be used for screening molecular libraries against pre-determined targets and for lead optimization.

## Sample Matrix

SPROX, DARTS, and LiP can be only performed using a cell/tissue lysate or homogenates as the added proteases cannot penetrate the plasma membrane, and chemical denaturants lyse the cells (Kuer et al., 2019). NanoBRET can be performed with intact cells and in cell lysates, but in particular, the experiments are run with intact cells. CETSA can be performed using intact or lysed cells and tissues as heating denatures the proteins regardless if they are inside or outside of the plasma membrane (Martinez Molina et al., 2013). This does not mean that it would not matter in which sample matrix CETSA experiments are run, quite contrary. First, when using intact cells as the sample matrix only those test compounds that have entered into the cell can cause an effect, unless the target is a surface receptor. Second, when performing experiments on lysed cells, all the biological processes should be considered non-active whereas in the intact cells the relevant biology is active, and cells are able to respond to treatment. In lysates, the proteins are not in their native microenvironment, and their natural ligands are not within physiological concentrations and also protein–protein interactions can be largely disrupted, depending on the buffer choice. Still, direct interactions between the ligand and protein are occurring and can be detected but might require the addition of the enzyme's natural substrate, for example, ATP or GTP (Vasta et al., 2018; Sridharan et al., 2019). In intact cells, for example, the proteins are in their native microenvironment, protein complexes are intact, and signaling cascades are activated or inactivated, thus making intact cells a suitable matrix for studying pathway effects besides the direct ligand–protein interactions. The data obtained from one matrix should be considered complementary to the other matrix but not excluded as some target interactions can be observed only in one of the matrices.

## Evolution of the CETSA Formats

### Melt Curve and Dose Response Curve

**FIGURE 2:**
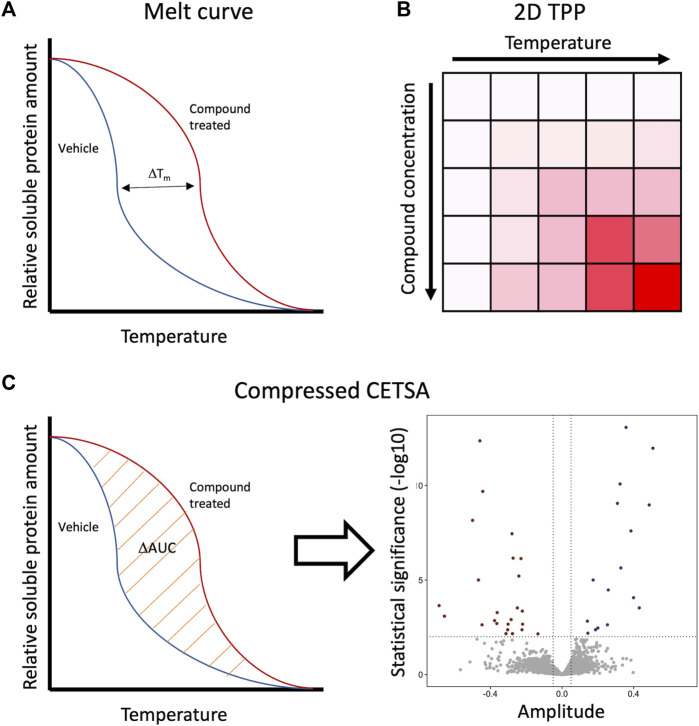
Ways to represent the data from different CETSA formats. **(A)** Melt curve represents the soluble protein amount as a function of temperature. Thermal shift magnitude is represented as ΔT_m_. **(B)** 2D TPP results are usually represented as a two-dimensional matrix, where the color intensity reflects the magnitude of ΔT_m_. Each temperature and compound concentration are plotted separately. **(C)** In compressed CETSA, protein average abundancy is measured, which is basically melt curves of AUC. These AUCs of the vehicle and compound treated samples are compared to each other and represented in a volcano plot as amplitude.

In CETSA, protein melt curves or dose response curves can be created, regardless of which detection method is used. In a protein melt curve experiment, the sample is treated with a saturating concentration of a ligand and aliquoted, and each aliquot is subjected to one step in a heat gradient. This is visualized in a graph which shows the soluble protein amount as a function of temperature (Figure 2A). The shift (destabilization or stabilization) scale in a melt curve does not indicate compounds’ potency only that there has been a compound–target interaction. To assess the potencies of different compounds, an isothermal dose response (ITDR) assay is required. In such an experiment, the sample is aliquoted, treated with a concentration series of the test compound, and then heated at a single temperature. It is advised to use the data from the melt curve assay to determine a suitable temperature for ITDR (Martinez Molina et al., 2013).

With WB and bead-based chemistry detection, which both rely on antibodies in the detection of the target protein, running a melt curve experiment prior to an ITDR is the usual experimental flow. As bead-based chemistry detection is relatively easy to automate, it is used in high-throughput CETSA experiments (Jafari et al., 2014; Almqvist 2016). In addition to screening for compound libraries, CETSA HT could be used for structure–activity relationship (SAR) studies, as suggested by Axelsson et al. (2016), Axelsson et al. (2018).

With MS, the melting behavior of the whole proteome, sometimes referred to as meltome, of the sample is investigated in one go (Savitski et al., 2014). This means that a melt curve can be extracted for each protein shifted by the test compound.

### 2D CETSA MS (Thermal Proteome Profiling)

Running a melt curve at each concentration of the dose response curve and vice versa is the way to get the most data out of the CETSA MS experiment. This method is referred to as 2D CETSA MS or as thermal proteome profiling (TPP). In addition to determining a shift in the melt curve, it determines a dose response curve for each temperature (Figure 2B). These dose responses make it typically easier to interpret the results, especially if the Tm shift in a protein is small. The drawback of this method is a large amount of a sample in the order of 10^8^ cells per experiment/test compound, the long running time on the MS instrument it requires, difficulty to perform automated searches for the results, or making statistical overviews (Savitski et al., 2014; Kuer et al., 2018).

### Compressed CETSA MS (PISA)

In 2019, first Liu et al. (2019) and a bit later Gaetani et al. (2019) demonstrated that pooling the samples from each heat step to a single sample is an effective way to decrease the amount of sample and MS instruments run time while preserving most of the information from the 2D format. Instead of representing the results in heat maps in 2D format, the melt curve data are compressed as ΔAUC, area under the curve, between the negative control and test compound in each concentration (Figure 2C). Pooling of the heat steps leads to the loss of information about so-called non-CETSA abundance effects, for example, changes in the expression level or degradation of the protein. Fortunately, this can be overcome by preparing a non-heated sample otherwise treated similarly to the corresponding CETSA sample and a non-heated sample treated only with the vehicle (Gaetani et al., 2019). Pooling together samples from a wide temperature range can make it hard to detect small ΔAUC shifts and proteins with non-sigmoidal melting patterns. To overcome this, the samples from the temperature gradient could be pooled in smaller pools of three temperatures instead of 10 temperatures. This idea was published by Li et al. (2020). They showed with simulated and experimental data that selecting only three temperatures can improve the resolution and help find unique shifters compared to pooled samples from a 10-temperature gradient. As expected, also some proteins detected with a 10-temperature gradient were not detected with the selected smaller temperature gradient (Li et al., 2020). Another way to reduce the sample load in CETSA is iTSA (isothermal shift assay), where the samples are treated only at the proteomes' median melting temperature (Ball KA et al., 2020). The most profound problem with this method is that only proteins shifting close to this one temperature can be detected.

## Increase in the Capacity in CETSA MS

Typically, the CETSA MS samples are labeled with an isobaric tandem mass tag (TMT) (Thompson et al., 2003) to enable multiplexing. At the time CETSA MS was introduced, 10-plex and 11-plex TMT mass tags were entering the market. The throughput increased significantly after 16-plex TMT was introduced in 2019 (Thompson et al., 2019). The efficiency of the 16-plex over the traditional 11-plex was recently reported (Zinn et al., 2021). In their study, Zinn et al. (2021) showed that when making an 8-point melt curve with 20 μM staurosporine, they were able to quantify about the same amount of proteins but detected an additional 5% significantly shifted kinases in ½ machine time with 16-plex TMT when compared to 11-plex.

Currently, the rate-limiting step in CETSA MS is the mass spectrometry run time. One researcher running CETSA experiments can produce four 16-plex TMT sets in 1 day. The next day the researcher can prepare all these samples for mass spectrometry, but acquiring the data on the mass spectrometer will take 4 days as acquiring data from one 16-plex TMT takes about 24 h. By dividing each 16-plex TMT for 13 sample slots with two vehicle controls and one positive control, it is possible to run three biological replicates of 52 compounds in 2 weeks using only one mass spectrometer. Utilizing this kind of setup, we screened the binding profiles of 192 compounds as proof of concept for the EUbOPEN project, which is a joint effort from the academia and industry to develop high-quality chemical tool compounds for 1,000 human proteins by the year 2025 (www.eubopen.org).

Obviously, this kind of throughput is nowhere near the CETSA HT or NanoBRET systems which can be conducted on several 384-well plates simultaneously. But these methods produce data only on the behavior of a single protein at a time, while compressed CETSA MS produces data from 7000–8000 simultaneously (Gaetani et al., 2019; Chernobrovkin et al., 2021; Zinn et al., 2021; Xu Y et al., 2021). Thus, the compressed CETSA MS (PISA) is a powerful tool, when assessing the melting behavior of the global accessible proteome.

## Selectivity, Co-Targets, and Off-Targets

Even though most of the drugs are designed to be as specific as possible, it is very rare that a chemical compound would only bind to one protein in a cell, especially when the concentration of the compound gets higher. Also, there are drugs on the market that are known to bind to several proteins, for example, palbociclib and midostaurin, for which the clinically relevant effects are resulting from the inhibition of more than one single kinase (Ramsay et al., 2018). In this kind of case, it would be justified to refer to these kinases as co-targets rather than off-targets.

Compound selectivity is usually studied by comparing their inhibitory capacity, binding affinity, or effective concentration between the target protein and its close homologues. Compounds are also screened against representative sets of other proteins mediating similar biological processes from different sub-families, for example, sets of protein kinases. Usually, these protein panels used, for example, in kinetic assays or TSAs are so vast that no individual cell type expresses all those proteins and thus gives a more systemic overview of the potential off-targets. On the other hand, these proteins are often not full-length proteins, but for technical reasons, protein domains lack the quaternary structure and post-translational modifications of the full-length protein. The proteins present in the lysate from cells and tissue are full length and have post-translational modifications, but these proteins are in a dilute environment lacking trace elements, and some protein complexes might be disrupted, and thus, some potential (off) targets might not be detected. Using intact cells as a profiling matrix considers/includes/reports on the permeability and efflux of the compound, which might differ between different cell types because of their differential transporter and efflux pump expression (Mandal et al., 2017). Because there is no perfect system it might be good to use several different approaches, for example, if working with kinases, first, different kinase families that are affected by the compound are screened quickly, and after this cell lines expressing these kinases are selected to confirm the results in this more biologically relevant system.

## Current Advances in CETSA MS

In this section, we will provide a limited set of examples of how researchers have utilized CETSA in their recent publications. It is good to note that we do not cover all the recent publications that have utilized CETSA.

### CETSA MS Helps Elucidate Biological Pathways and Detailed MOA

CETSA conducted with intact cells together with proteome-wide mass spectrometry readout can provide us information on the activated biological pathways, thus helping to solve the detailed mode of action (MOA) of the test compound.

Liang et al. (2021) described beautifully the detailed MOA for a cancer drug 5-fluoro uracil (5-FU) which was solved by using proteome-wide CETSA. In their study, the effect of 5-FU on thymidylate synthase (TYMS), the known primary target for 5-FU, is confirmed. Interestingly, most of the interaction changes they detected were not associated with the MOA of 5-FU on TYMS. When they studied 5-FU–resistant cell lines, they found that TYMS was still fully affected by 5-FU, indicating that inhibition of TYMS is not essential for the cytotoxicity of the drug. Instead, they discovered that 5-FU also affects proteins involved in RNA modification and interactions in normal cells but not in 5-FU–resistant cells. The results led the authors to suggest the use of some of the proteins highlighted in the study as biomarkers for assessing 5-FU efficacy in human tumors (Liang et al., 2021).

A common MOA for three anti-cancer compounds RITA (reactivation of p53 and induction of tumor cells apoptosis), aminoflavone (AF), and oncrasin-1 (Onc-1) was revealed utilizing CETSA MS (Peuget et al., 2020). As the name suggests, the MOA of RITA has been hypothesized/suggested to be through reactivation of p53, but this has lately been questioned (Wanzel et al., 2016). The MOA of AF and Onc-1 were unclear, but they induced highly similar cell responses (Peuget et al., 2020). Using TPP, these three compounds were shown to target mRNA processing and transcription. In further experiments RITA, AF, and Onc-1 were demonstrated to inhibit these processes under increased oxidative stress, which makes them highly cancer cell–specific, again showing the potent, agnostic profiling strength of CETSA.

In a third example, combining TPP, quantitative proteomics, metabolomics, and *in silico* molecular docking, a novel protein target and MOA were revealed for the metabolite of the plasticizer di(2-ethylhexyl) phthalate and monoethylhexyl phthalate (MEHP) (Xu T. et al., 2021). In the TPP experiment conducted with the cell lysate, 74 proteins were shifted by MEHP, and in molecular docking, MEHP bound to all 36 proteins with a published crystal structure. When all the multi-omics data had been integrated into metabolite–gene and protein–protein interaction networks, MEHP seems to affect metabolites and proteins important in the cell cycle transition from G1 to S phase. MEHP was confirmed to hinder cell transit from the G1 phase to S phase with a flow cytometry assay (Xu T. et al., 2021).

### Combining CETSA With Other Target Deconvolution Methods

Recently researchers from Pfizer have introduced a compressed format of SPROX. They pooled samples from the denaturant gradient as one sample and compared it to the compressed CETSA MS (Xu Y. et al., 2021). This study shows that compressed CETSA MS and SPROX achieve ∼ 1,56 and ∼1,62 higher protein coverage, respectively, and ∼10 higher throughput than previous formats. SPROX was able to pick up 68% of the proteins detected in CETSA MS, which is due to its limitation to detect only methionine peptides. On the other hand, SPROX requires only a third of the mass spectrometry machine time that CETSA requires. Most importantly the protein coverage of these methods is not fully overlapping, and in the study, they both provided a significant amount of unique hits for staurosporine. Thus, the data these methods provide are more complementary than competing (Xu Y. et al., 2021). Combining these two methods together could prove useful, especially in target deconvolution and pointing to the compound binding site.

In a very recent study, describing a novel CDK9 inhibitor, the researchers have combined chemoproteomics and kinase affinity tools, i.e., Kinobeads, CETSA MS, and LiP. The article describes how these orthogonal methods create together convincing evidence of the specificity of the novel inhibitor and how the methods complement each other (Hendricks et al., 2022).

### Cell Surface Thermal Proteome Profiling

Many drugs target receptors that are located on the cell surface. To study *in situ* target engagement of these receptors, live cells are needed, which limits the selection of the test methods. NanoBRET is one of the methods that can be used in such cases, and in 2015, the first CETSA application to study the target engagement of transmembrane proteins was introduced (Reinhard et al., 2015) and was extended to a broad application to multipass transmembrane proteins in 2019 (Kawatkar et al., 2019). These methods utilize detergents, but a detergent-free CETSA MS method has been published just recently (Kalxdorf et al., 2021). Kalxdorf’s application relies on enriching biotinylated cell surface proteins. The published workflow is suitable only for suspension cells (Kalxdorf et al., 2021), but with some modifications, they could also be applied to adherent cell lines. The authors hypothesize that trafficking and receptor uptake remove proteins from the plasma membrane and can affect the results by producing false negatives. The internalization of the target protein can be blocked by inhibiting or removing the key component of the internalization machinery, like in the case of CXCR4 (Kalxdorf et al., 2021). But this is possible only if the identity of the target and the internalization route are known.

### Single Cell CETSA

One of the most interesting novel applications for CETSA was introduced in 2021 (Osman et al., 2021)—CETSA performed with readout on a single-cell level. This application is exciting as it reduces the number of cells needed for a single experiment from millions to just a few hundred. This makes it possible to perform experiments with precious patient materials like fine needle aspiration (FNA) biopsies. CETSA has previously been applied for FNA samples using at least 10,000 live cells, but with such a cell amount, it is hard, for example, to produce replicates, test different drugs, or test dose/concentration responses, which the authors bring up in an article from 2019 (Langebäck et al., 2019). Osman et al. (2021) conducted the CETSA step in microcentrifuge tubes, followed by injection into a microchip. In the chip, the cells were directed to specific chambers and optically lysed with a laser. Each chamber of the chip contained an immobilized capture agent; in this case it was DNA, which pulled down the soluble target protein. The bound proteins were labeled with fluorescent-labeled antibodies and detected by total internal reflection fluorescence (TIRF) microscopy. It is possible to count the number of bound copies of the target protein from the TIRF images and to compare the detected copy numbers between treated and untreated samples. The study tested the ability of the microchip-based single cell CETSA in detecting the target engagement of three test compounds. Indeed, they found two compounds to stabilize the target protein and one to destabilize it. The results were verified for the two stabilizers, but not for the destabilizing compound, by using whole-cell Western blot–based CETSA. Also, others have suggested using fluorescence imaging as a detection method for CETSA performed on 96- or 384-well plates to decrease required cell amounts, but they are not truly single-cell formats (Axelsson et al., 2018; Massey, 2018). The downfall in all these fluorescence imaging approaches is that they need an antibody that can specifically distinguish between the folded and aggregated form of the protein.

The development of machine power, resolution, and process time over the years has led to the point that single-cell mass spectrometry is emerging as a novel technology (Slavov 2021). Combining single cell mass spectrometry with single-cell CETSA is something that is investigated, and if successful, it will create a whole new field of applications for the CETSA method.

## Discussion

Over the years, CETSA has established its position as one of the key methods in target engagement studies. To create a safe drug or a good chemical probe, it is not only enough to know that the molecule binds to the intended target but also to which other proteins or off-targets it binds to. Mass spectrometry detection is the best way to detect the most proteins in the shortest time; thus, it is utilized for most target deconvolution studies. From the methods based on the detection by MS, CETSA covers the largest portion of proteome compared to SPROX or DARTS/LiP (Kaur et al., 2018), but these methods do not cover all the same parts of the proteome and can reveal novel targets (Xu Y et al., 2021). Thus, these orthogonal methods do not provide only confirmation of the findings but also novel information about the properties of the studied item.

Combining an HT assay with CETSA MS could prove to be a beneficial tool for more physiologically relevant SAR. First, a large SAR library could be run to rank out the non- and low-binding test compounds followed by, for example, compressed CETSA MS to determine the co-targets and off-targets of the remaining test compounds.

Utilizing intact cells as the study matrix in CETSA gives an additional benefit of observing the secondary, biological effects of the study item. Combining these data together with conventional proteomics, RNA transcriptomics, and metabolomics studies can provide valuable information on the systemic mode of action for the study item. When the study system is updated from a living cell to an artificial tissue model, it might be even more relevant to estimate the compound´s true biological effectiveness and potential adverse outcomes in humans. Providing a better biological understanding on how the chemicals, intended to become drugs, work in a living cell, tissue models, and *ex vivo* tissues will help create more efficient and safer drugs in the future.
